# The Institutional Worker

**Published:** 1911-12-16

**Authors:** 


THE
### INSTITUTIONAL WORKER
**Being a Special Supplement to "The Hospital"**

### Editor's Notices.

**Contributions:**
Contributions should be written, or preferably typed, on one side of the paper only, and all articles sent in are accepted upon the distinct understanding that they are forwarded to The Hospital only.

The Editor cannot undertake to return MSS. not used, but when a stamped directed envelope is enclosed the MSS. may be returned if a special request is made.

Accepted articles and paragraphs of news will be paid for after publication at the scale rate.

**Address:**

To prevent delay all contributions and letters on editorial business must be addressed exclusively to the Editor, "The Hospital," 29 Southampton Street, Strand, London, W.C. It is important that this regulation shall be strictly observed.

**Correspondence:**

Correspondence on all subjects is invited. The name and address of correspondents must be given as a guarantee of good faith, but not necessarily for publication.

**Special Articles:**

Special articles are invited, and questions, inquiries, and paragraphs upon all matters relating to the work, administration, and management of general, special, mental, fever, cottage and convalescent hospitals, sanatoria, homes, institutions, societies and organisations for the treatment or care of the sick, injured and dependents of all classes. Every matter affecting the interests and welfare of the staff of all grades working in these institutions will receive special consideration.

**Administrative Medicine, Research and Items of News:**
Special terms will be paid for approved articles on subjects in this wide field by experts who have made some section of it their special study and interest. We wish to give prominence to every movement tending to advance accuracy of diagnosis, efficiency in treatment, and the development of modern methods to eradicate disease and promote the welfare of the suffering.

**A Bureau of Information:**
We invite inquiries and applications for information and help in providing for that numerous class of sufferers who require special care or house-room which they cannot obtain in their own homes or secure for themselves. We cannot, however, prescribe or recommend practitioners.

**Books for Review:**

Publishers are particularly requested to send advance proofs of any new books of importance whenever possible, as the Editor has made arrangements to publish immediate reviews on a new plan.

**Photographs, Plans, Blocks, and Illustrations:**
It is requested that wherever possible MSS. may be accompanied by illustrations in any of the above forms. The name of the sender and of the article to which it belongs should be written on the back of each photograph, drawing, or block for purposes of identification.

**Local Papers:**
Newspapers containing reports or news paragraphs should be marked and addressed to the Sub-Editor.

**The Coupon System:**

A coupon will be found at the bottom of the third inside page of the cover each week. This coupon must be attached to every question or inquiry to which an answer is desired in The Hospital.

### Manager's Notices.

Letters relating to the publication, sale, and advertising departments of The Hospital must be addressed to the Manager, "The Hospital," The Hospital Building, 28 and 29 Southampton Street, Strand, London, W.C.

**Subscriptions** which may begin at any time, are payable in advance. Cheques and Post-Office orders, crossed London County and Westminster Bank, Covent Garden branch, should be made payable to the Manager of The Hospital and addressed to him at The Hospital Building, 28 and 29 Southampton Street, Strand, London, W.C.

**Advertisements:**
All advertisements for The Hospital must reach the Manager not later than Wednesday morning in each week. For scale of charges see pages v and 2.

**Classified Advertisements:**
All small advertisements will be classified, for this section of the paper is widely read and of special interest. We wish to encourage those wanting appointments who are attracted to institutional work, and therefore offer them the reduced rate of *twenty-one words and under for* 1s., and *for every ten and part of ten words beyond*, 6*d*.

### POINTS FOR INSTITUTION AUTHORITIES.

The economical management of an Institution depends very greatly upon purchasing supplies in the cheapest market.

It is often impossible locally to secure the highest quality at the lowest prices.

In consequence, a section will be reserved in this part of the paper for announcements of Institutions inviting Tenders foı Contracts.

This will enable Authorities of Institutions to secure tenders for supplies from all over the country, and ensure purchases at the lowest possible prices consistent with quality.

The charge for Tenders is 6s. per inch, and for this small outlay many pounds may be saved on supplies of all descriptions.

**Special Rates** will be quoted for those institutions which agree to send all their vacancy, contract, and public notices to The Hospital each year, so as to make it a file of such announcements for ready reference.

### POINTS FOR TRADE ADVERTISERS.

The Hospital is the organ of Institutions, whether of a Voluntary Character or those under Local Government, Poor Law and Municipal Authorities.

It is read by all Executive Officials and those responsible for the Construction, Management, and Equipment of Public Establishments.

It is also the Journal of the worker engaged in the Medical, Health and Preventive services.

There is no medium more appropriate and effective in the promotion of business in these directions than The Hospital.

### POINTS FOR INSTITUTIONAL WORKERS.

A very large number of Appointments Vacant appear every week in the columns of The Hospital.

If you cannot find a position suited to your requirements, make your wishes known through our Situations Wanted Column.

### APPOINTMENTS VACANT.

### Poor-Law Vacancies.

### Appointments Vacant.

Assistant Female Relieving Officer (£75), Norwich Guardians. Mr. E. J. W. Huggins, C. to G., Norwich.

Assistant Infirmary Steward (30s. weekly, non-resident), King's Norton Union. Mr. R. J. Curtis, C. to G., Guildhall Buildings, Birmingham. Dec. 18.

Assistant Master (£40), Northampton Union. Mr. W. Fawkes, C. to G., Northampton. Dec. 28.

Assistant Matron (£30), Romford Union. Mr. C. Bloomfield, C. to G., Romford. Dec. 18.

Assistant Matron (£40), Southampton Union. Mr. A. J. Walden, C. to G., Southampton. Dec. 16.

Assistant Matron (£25), Whitehaven Union. Mr. J. Bowly, C. to G., Whitehaven. Dec. 18.

Assistant Nurse (£25), Whitehaven Union. Mr. J. Bowly, C. to G., Whitehaven. Dec. 18.

Assistant Nurse (£20), Kingsclere Union. Mr. J. Barnes, C. to G., Kingsclere. Dec. 20.

Assistant Nurse (£25), Hereford Union. Mr. R. Moore, C. to G., Hereford. Dec. 22.

Assistant Nurse (£25), Bedford Union. Mr. W. Payne, C. to G., Bedford. Dec. 23.

Assistant Nurse (£25), King's Lynn Union. Mr. R. C. Coulton, C. to G., King's Lynn.

Assistant Nurse (£25), Wortley Union. Mr. J. Morton, C. to G., Sheffield.

Assistant Nurse (£25), Glanford Brigg Union. Mr. F. C. Hett, C. to G., 11 Bigby Street, Brigg. Dec. 20.

Assistant Nurses (£22), Sevenoaks Union. Mr. F. H. Vibart, C. to G., Bank Chambers, Sevenoaks. Jan. 1.

Assistant Warrant Officer (£80), Pontypridd Union. Mr. W. Spickett, C. to G., Pontypridd. Dec. 29.

Charge Nurse (£30), Dudley Union. Mr. E. Allen, C. to G., Dudley. Dec. 18.

Charge Nurse (£30), South Shields Union. Mr. J. W. Coulson, C. to G., Barrington Street, S. Shields. Dec. 16.

Charge Nurse (£32), Cheltenham Union. Mr. J. Meek, C. to G., Swindon Road, Cheltenham. Dec. 20.

Charge Nurse (£32), Luton Union. Mr. W. Austin, C. to G., 7 George Street West, Luton. Dec. 19.

Female Attendant (£20), Tendring Union. Matron, Tendring Infirmary, Weeley, S.O.

Female Imbecile Attendant (£25), Prestwich Union. Mr. E. W. Ogden, C. to G., Cheetham Hill Road, Manchester. Dec. 18.

Foster-Mothers (£25 and £22 10s.), Birkenhead Union. Mr. J. Carter, C. to G., Birkenhead. Dec. 21.

Foster-Mother (£22), Cardiff Union. Mr. A. J. Harris, C. to G., Cardiff. Dec. 22.

Home Sister and Second Assistant Matron (£50), South Manchester Guardians. Mr. D. S. Bloomfield, C. to G., All Saints', Manchester. Dec. 19.

Junior Assistant Nurse (£15), Malling Union. Mr. F. J. Allison, C. to G., West Malling. Dec. 20.

Junior Assistant Nurses (two) (£22), Totnes Union. Mr. F. K. Windeatt, C. to G., 19 High Street, Totnes. Dec. 28.

Male Attendant (£26), Islington Guardians. Master of the Workhouse, St. John's Road, Upper Holloway, N.

Male Imbecile Attendant (£26), Lincoln Union. Mr. W. B. Danby, C. to G., Lincoln.

Male Ward Attendant (£25), Cheltenham Union. Mr. J. Meek, C. to G., Cheltenham.

Master and Matron (£100), Grimsby Union. Mr. J. F. Wintringham, C. to G., Great Grimsby. Jan. 1.

Master's Assistant Clerk (£25), Hackney Union. Master of the Workhouse, Sidney Road, Homerton, N.E.

Midwife (£32), Greenwich Union. Matron of the Infirmary, Vanbrugh Hill, East Greenwich. Dec. 18.

Midwife (£35), Marylebone Guardians. Matron of the Workhouse, Northumberland Street, Marylebone Road, W.

Night Sister (£40), Brighton Guardians. Mr. B. Burfield, C. to G., Parochial Offices, Brighton. Jan. 2.

Night Superintendent (£40), Ecclesall Bierlow Union. J. E. Moulding, C. to G. Union Offices, The Edge, Sheffield. Jan. 2.

Night Superintendent and Masseuse (£38), Brentford Union. Mr. W. Stephens, C. to G. Union Offices, Isleworth, W. Jan. 2.

Nurse (£32), Leigh Union. Mr. A. H. Hayward, C. to G., Union Offices, Leigh, Lancs. Dec. 20.

Nurse (£30), Ticehurst Union. Mr. J. Chase Edwards, C. to G., Union Offices, Wadhurst, Sussex. Dec. 16.

Nurse (£35), Tendring Union. Matron, Tendring Infirmary, Weeley, S.O.

Nurses (£30), Gateshead Union. G. Craighill, C. to G. Poor Law Union Offices, Gateshead. January 6.

Nursery Attendant (£25), Marylebone Guardians. Matron of the Workhouse, Northumberland Street, Marylebone Road, W.

Probationer (£13), West Ham Union. Mr. T. Smith, C. to G., Leytonstone, N.E.

Probationer (£12), Kettering Union. Mr. E. R. Lane, Acting C. to G., Poor Law Offices, George Street, Kettering. Dec. 28.

Probationer (£10), North Bierley Union. Mr. W. G. Cooper, C. to G., 4 Town Hall Street, Bradford. Dec. 30.

Seamstress and Relief Foster-Mother (£25), Darlington Union. Mr. C. H. Leach, C. to G., Darlington. Dec. 16.

Schoolmaster (£65), West Ham Union. Mr. T. Smith, C. to G., Leytonstone, N.E. Dec. 20.

Schoolmistress (£70), Brighton Guardians. Mr. B. Burfield, C. to G., Brighton. Dec. 20.

Staff Nurse (£24), Holborn Guardians. Matron, Workhouse, 1a Shepherdess Walk, N.

Superintendent of Laundry (£35), Marylebone Guardians. Matron of the Workhouse, Northumberland Street, Marylebone Road, W.

Superintendent Nurse (£40), Windsor Union. Mr. P. Lovegrove, C. to G., 3 Park Street, Windsor. Dec. 18.

Tailoring Attendant (£35), St. Pancras Guardians. Mr. J. E. P. Hall, C. to G., Town Hall, Pancras Road, N.W.

Ward Sister (£30), Woolwich Union. Mr. T. Cutter, C. to G., Union Offices, Woolwich. Dec. 18.

Ward Sister (£30), Southampton Union. Mr. A. J. Walden, C. to G., Southampton. Dec. 16.

Ward Sister (£30), Bermondsey Guardians. Matron, Infirmary, Lower Road, Rotherhithe.

### Situations Vacant.

Assistant Laundress (18s. weekly, non-resident), Islington Guardians. Matron of the Infirmary, Highgate Hill, Upper Holloway, N.

Cook (£25), Northallerton Union. Mr. W. Fowle, C. to G., Northallerton. Dec. 20.

Cook, etc. (£30), Huddersfield Union. Mr. E. A. Rigby, C. to G., Huddersfield.

Female Cook (£30), Ticehurst Union. Mr. J. C. L. Andrews, C. to G., Wadhurst. Dec. 16.

Hospital Cook (£27), Burton-on-Trent Union. Mr. C. F. Chamberlin, C. to G., Burton-on-Trent. Dec. 18.

Laundress (£28), Royston Union. Mr. A. Sharpe, C. to G., Royston, Herts. Dec. 19.

Laundress (£25), Holbeach Union. Mr. S. S. Mossop, jun., C. to G., Holbeach.

Porter and Cook (married couple) (£20 each), Walsingham Union. Mr. R. S. Butcher, C. to G., Fakenham. Dec. 16.

Stoker and Washing-machine Attendant (£32 12s.), Islington Guardians. Headmaster of the Schools. Hornsey Road, N.

### Institutional and Administrative Vacancies.

### Appointments Vacant.

Matron (£40), Brixham Cottage Hospital. Apply the Matron. Dec. 19.

Matron (£60), Putney Hospital. Secretary, 198 Upper Richmond Road, Putney, S.W. Dec. 31.

Sister (£30), Birmingham General Hospital. Apply Matron.

### Municipal Vacancies.

### Appointments Vacant.

Assistant Nurses (£25), Warrington Borough. Superintendent of Nurses, Isolation Hospital, Warrington.

Clerk of Works (County Buildings) (2½ guineas weekly), Wilts. Standing Joint Committee. Mr. J. C. Powell, County Surveyor, Trowbridge. Dec. 18.

County Finance Clerk (£200), Brecon C.C. Mr. H. F. W. Harries, Clerk to the Council, Brecon. Dec. 19.

Health Visitor (£80), Wolverhampton Borough. Medical Officer of Health, Red Lion Street, Wolverhampton. Dec. 27.

Health Visitor (£75), Borough of Chesterfield. Mr. J. Middleton, Town Clerk, Chesterfield. Dec. 16.

Nurse (£26), Llandaff and Dinas Powis Rural District Council Isolation Hospital. Mr. M. Warren, Clerk. Park House, Cardiff. Dec. 27.

Probationer (£12), Southgate U.D. Council. Matron, Isolation Hospital, Palmers Green, N.

### Situations Vacant.

Porter (£25), Farnham Joint Isolation Hospital. Mr. E. Crundwell, Clerk to the Committee, Farnham. Dec. 20.

### Miscellaneous Vacancies.

Dispenser (£35), Croydon Mental Hospital, Upper Warlingham, Surrey. Medical Superintendent.

Nurse (£70), York Education Committee. Mr. J. H. Mason, Secretary, Education Offices, Clifford Street, York. Dec. 27.

Nurse (£80), Cheltenham Education Committee. Secretary, Education Offices, Cheltenham.

School Nurse (£70), City of York Education Committee. Mr. J. H. Mason, Secretary, Education Offices, Clifford Street, York. Dec. 27.

School Nurse and Health Visitor (£85), Borough of Bromley. Mr. F. Thackeray, Town Clerk, Morley, Yorks. Dec. 28.

Superintendent, Steward, Engineer, Matrons, Nurses, Attendants. The National Institutions for Persons requiring Care and Control. Secretary. (See advt.).

Veterinary Inspector, Wilts C.C. Mr. R. W. Merriman, Clerk to the Council, Trowbridge. Dec. 31.

### Situations Vacant

Cook (£34), Stafford County Asylum. Medical Superintendent of the Asylum, Stafford.

### APPOINTMENTS, RESIGNATIONS, &c.

### Poor Law Appointments.

Alexander, Mr. H. G., Third Resident Assistant Medical Officer at the Infirmary, St. Marylebone Parish.

Attwater, Mr. H. L., Resident Assistant Medical Officer at the Infirmary, Southwark Union.

Bonora, Miss B. O., Pupil Day Nurse, Chelsea Parish.

Brander, Mr. W., Medical Superintendent of the Infirmary, Homerton Workhouse and Children's Homes, etc., Hackney Union.

Bratt, Miss M., Matron of the Cottage Home, Chorley Union.

Buchanan, Mr. D. Y., Resident Assistant Medical Superintendent at the Infirmary, Croydon Union.

Busby, Mr. G. W., Master of the Workhouse, Thornbury Union.

Busby, Mrs. E. A., Matron of the Workhouse, Thornbury Union.

Gregorson, Mr. A. W., First Resident Assistant Medical Officer at the Infirmary, Edmonton Union.

